# The association between perceived social support and self-management behaviors in adolescents and young adults with inflammatory bowel disease: the chain mediating role of basic psychological needs and anxiety/depression

**DOI:** 10.3389/fpsyg.2025.1483021

**Published:** 2025-02-05

**Authors:** Yangfan Zhu, Yueyue Chen, Yuman Tang, Xin Zhang, Qiao Shen, Fei Li, Hao Wang, Xianlan Zheng

**Affiliations:** ^1^Department of Nursing Children's Hospital of Chongqing Medical University, National Clinical Research Center for Child Health and Disorders, Ministry of Education Key Laboratory of Child Development and Disorders, Chongqing Key Laboratory of Pediatric Metabolism and Inflammatory Diseases, Chongqing, China; ^2^Department of Gastroenterology, Chongqing General Hospital, Chongqing University, Chongqing, China; ^3^Mental Health Education and Counseling Center, Chongqing Institute of Foreign Studies, Chongqing, China

**Keywords:** inflammatory bowel disease, adolescents, young adults, self-management, social support, anxiety, depression

## Abstract

**Introduction:**

The incidence of inflammatory bowel disease (IBD) has been increasing, with adolescents and young adults being the peak age of onset. Self-management behaviors were demonstrated to enhance remission and quality of life, yet the mechanisms influencing self-management behaviors remained under-explored. Perceived social support is crucial to self-management behaviors, alongside the roles of basic psychological needs, anxiety, and depression.

**Methods:**

We conducted a two-center cross-sectional survey in China from July to August 2024 via convenience and snowball sampling to investigate how these variables influence self-management behaviors. Data were collected utilizing the structured self-report questionnaires. Mediating effects were analyzed using the bootstrap method.

**Results:**

A total of 183 adolescents and young adults with IBD (male: 71.58%), aged 13 to 24 years old (*M* = 20.33, *SD* = 3.03), were included in the analysis. The research findings include the following points: (1) perceived social support positively predicted self-management behaviors (β = 0.767, *P* < 0.001); (2) perceived social support affected self-management behaviors through chain mediation involving basic psychological needs and anxiety/depression.

**Conclusion:**

Clinical practitioners should enhance social support for adolescents and young adults with IBD and improve their perceptions of such support, fulfill basic psychological needs, and alleviate anxiety and depression to promote effective self-management behaviors.

## 1 Introduction

IBD, including ulcerative colitis (UC), Crohn's disease (CD), and unclassified types, is a chronic autoimmune disease of the gastrointestinal tract (Rubalcava and Gadepalli, 2021). The global incidence of IBD has been continuing to rise, with adolescents and young adults being the most prevalent population for this disease (Rubalcava and Gadepalli, 2021). In China, the prevalence of IBD was the highest in Asia (Ng et al., 2013), with an incidence rate of 10.04 cases per 100,000 person-years in 2016 (Xu et al., 2023). The clinical features of IBD include extreme fatigue, recurrent diarrhea, rectal bleeding, abdominal cramps, and growth retardation (Rubalcava and Gadepalli, 2021), which cause great suffering to adolescents and young adults patients (Barned et al., 2022).

Self-management behaviors (SMB) refer to actions adopted by patients to maintain and improve their health status (Miller et al., 2015). Self-management behaviors involves medical, emotional, and role management, focusing on health behaviors, coping with stress, and maintaining daily roles despite the disease. The adolescents and young adults phase is a critical transition period from childhood to adulthood, and self-management is essential for facilitating this process (White and Cooley, 2018). Research indicated that SMB could prolong remission in adolescents and young adults with IBD, which in turn could enhance their quality of life (Seyedian et al., 2019). Conversely, a lack of effective SMB could exacerbate symptoms and increase the risk of complications (Seyedian et al., 2019). Alarmingly, the status of SMB in adolescents and young adults with IBD was not optimistic (Krauthammer et al., 2020; Wu et al., 2022). Under the bio-psycho-social medical model, the influence of psychological factors on patients' SMB has been increasingly emphasized (Miller et al., 2015). Most previous studies have focused primarily on describing the status of self-management in this group (Krauthammer et al., 2020; Wu et al., 2022), with an insufficient exploration of the underlying psychological mechanisms. Therefore, this study aimed to explore the mechanisms of SMB in adolescents and young adults with IBD, aiming to provide insights into effective clinical interventions for SMB.

Expanding social relationships is also essential for adolescents and young adults (Sawyer et al., 2018). Research (Roberts et al., 2021) suggested that a lack of peer, family, and social support may contribute to the social isolation of adolescents and young adults with IBD. According to the buffer theory of social support (Miloseva et al., 2017), support from social relationships is an essential protective factor when suffering from psychological problems. Compared with actual social support, perceived social support is more likely to benefit individuals' psychological wellbeing, which refers to their expectations and subjective evaluations of the support they may receive (Geng et al., 2018). Research showed that the higher level of perceived social support among adolescents and young adults with IBD was associated with increased motivation to generate SMB (Kamp et al., 2019a).

Self-determination theory suggests that human motivation to adopt or refrain from behaviors is influenced by the satisfaction of basic psychological needs, including autonomy, competence, and relatedness (Ryan and Deci, 2000). Autonomy refers to the desire to be the origin or source of one's behavior. Competency involves a perception of confidence and effectiveness in one's actions. Relatedness is the desire to feel connected to others. Whether adolescents and young adults with IBD adopt SMB was influenced by basic psychological needs: previous investigations revealed that adolescents and young adults with IBD were overprotected by their parents (Tan and Ong, 2020), and their need for autonomy may be unmet; their lack of confidence in SMB (Cruz et al., 2024) also made it possible that their need for competence was unmet; furthermore, they were affected by peer victimization and the stigma of their illnesses (Roberts et al., 2021), which may result in the need for relatedness being unmet. It was reasonable to infer that unmet basic psychological needs influenced the poor SMB status of adolescents and young adults with IBD. Meanwhile, relationships within an adolescent's environment were essential for fulfilling basic psychological needs. Research (Chen et al., 2019) indicated that greater perceived social support corresponded to higher levels of basic psychological needs satisfaction, establishing a positive relationship between perceived social support and basic psychological needs. Thus, we were justified in proposing the first hypothesis of this study:

*H1*: perceived social support may positively affect SMB in adolescents and young adults with IBD through the positive mediation of basic psychological needs.

Changes in social roles can affect mental health of adolescents and young adults (Sawyer et al., 2018). Anxiety and depression are prevalent among adolescents and young adults with IBD (Mules et al., 2022). Research (Cao et al., 2023) showed that behavioral changes resulting from chronic anxiety and depression could lead to a lack of SMB motivation. The influence of anxiety and depression as significant variables in SMB should not be underestimated. Anxiety and depression often coexist, with ~85% of depressed patients experiencing significant anxiety and 90% of those with anxiety also showing depressive symptoms (Tiller, 2013). Therefore, two independent mediation models would be developed to analyze anxiety and depression as mediating variables in this study. Meanwhile, the higher the degree of perceived social support, the lower the likelihood of anxiety and depression in patients with IBD (Slonim-Nevo et al., 2018). Therefore, it was reasonable to propose the second hypothesis of this study:

*H2*: perceived social support may affect SMB in adolescents and young adults with IBD through the negative mediating role of anxiety/depression.

According to self-determination theory, the satisfaction of basic psychological needs could also influence mental health outcomes (Ryan and Deci, 2000). Previous research showed that basic psychological needs in adolescents and young adults was negatively correlated with anxiety (Liu et al., 2022) and depression (Vandenkerckhove et al., 2021). Therefore, it was reasonable to propose that basic psychological needs and anxiety/depression may collectively function as chain mediators between perceived social support and SMB. In summary, perceived social support may positively influence SMB, with both variables being intricately linked to the satisfaction of basic psychological needs. Furthermore, perceived social support and basic psychological needs may impact levels of anxiety and depression, which, in turn, could affect an individual's SMB. Consequently, we were warranted in proposing the third hypothesis:

*H3*: perceived social support may affect SMB in adolescents and young adults with IBD through the chain mediation involving basic psychological needs as a positive mediator and anxiety/depression as a negative mediator.

## 2 Methods

This study used a cross-sectional survey from July 2024 to August 2024 with a convenience sampling method. We invited adolescents and young adults with IBD attending two tertiary hospitals in Chongqing, China, to fill out questionnaires via an electronic questionnaire. Additionally, a snowball sampling method was implemented to increase the geographic diversity of the sample, encouraging participants who completed the questionnaire to forward it to adolescents and young adults with IBD whom they knew via social media software. To ensure the quality of the recovered questionnaires, we set an IP address to fill out the questionnaire only once, and all questions were required to prevent duplicate and incomplete information questionnaires.

### 2.1 Participants

The inclusion criteria were as follows: ①Confirmed diagnosis of IBD according to the Chinese guidelines for the diagnosis of IBD in adults and children (Chinese Medical Association, 2018, 2019). ②Age between 13 and 24 years, according to the World Health Organization's definition of Asian adolescents and young adults as individuals aged 10–24 years (World Health Organization, 2022), and the American Academy of Pediatrics' recommendation to introduce self-management conception around age 13 (White and Cooley, 2018) ③Knowledge of one's disease status. ④Ability in reading and comprehension. ⑤Understanding the purpose of the study and the ability to provide informed consent for participation. The exclusion criteria were as follows: ①Presence of perioperative and/or concomitant tumors. ②Diagnosis of a mental illness requiring psychiatric intervention.

### 2.2 Measurement of variables

#### 2.2.1 Demographic information

Demographic information included age, gender, residence, annual household income, educational background, disease type, disease duration, surgical history related to IBD, and the categories of medications currently being used.

#### 2.2.2 SMB

The Self-Management Behavior Scale for patients with IBD, developed by a Chinese scholar (Shang et al., 2019) was utilized to evaluate participants' SMB status. The scale consists of 36 items grouped into seven dimensions: medication management, diet management, disease monitoring, emotion management, exercise management, daily life management, and resource utilization. The responses were rated on a five-point Likert scale ranging from 1 (never) to 5 (always), with higher scores reflecting better SMB performance. The scale demonstrated a Cronbach's α coefficient of 0.945 in the original study and 0.941 in this research, indicating high internal consistency. This scale has been extensively employed by researchers to assess SMB among patients with IBD (Lu et al., 2024).

#### 2.2.3 Perceived social support

We utilized the Chinese version of Perceived Social Support Scale (Huang et al., 1996), developed by Blumenthal et al. (1987), to assess the level of perceived social support of the participants. The scale comprises 12 items categorized into three dimensions: family support, friend support, and other support. Responses were measured on a seven-point scale, ranging from 1 (strongly disagree) to 7 (strongly agree), with higher scores indicating greater levels of perceived social support. The Cronbach's α coefficient of 0.88 was in the original study and 0.943 in this study. The Chinese version has been widely adopted (Lin et al., 2024).

#### 2.2.4 Basic psychological needs

This study utilized a version of the Basic Psychological Needs Scale revised by Du et al. (2020). This version was based on the original one created by Sheldon et al. (2001), which was rooted in self-determination theory. The scale comprises nine items distributed across three dimensions: autonomy, competence, and relatedness. Responses were measured on a seven-point scale, ranging from 1 (strongly disagree) to 7 (strongly agree), with higher scores indicating greater fulfillment of basic psychological needs. The scale demonstrated good internal consistency, as evidenced by Cronbach's α coefficient of 0.86 in the original research and 0.941 in this study. Furthermore, the scale has been widely adopted in studies assessing basic psychological needs satisfaction among adolescents (Zhou and Wang, 2023).

#### 2.2.5 Anxiety

The Generalized Anxiety Disorder seven-item scale (GAD-7) was employed to assess participants' anxiety levels over the past 2 weeks. This scale consists of seven items and is scored on a four-point scale ranging from 0 (hardly ever) to 3 (almost every day), with higher scores indicating greater levels of anxiety. The GAD-7 has been widely utilized in clinical practice (Toussaint et al., 2020). In this study, the Cronbach's α coefficient for the GAD-7 was 0.937.

#### 2.2.6 Depression

For the assessment of participants' depression levels over the past 2 weeks, we utilized the Patient Health Questionnaire-9 (PHQ-9). This scale includes nine items and is scored similarly on a four-point scale from 0 (hardly ever) to 3 (almost every day), with higher scores reflecting increased levels of depression. The PHQ-9 has been used as a trusted measure of depressive symptoms in clinical settings (Ye et al., 2020). In this study, the Cronbach's α coefficient for the PHQ-9 was 0.920.

### 2.3 Data analysis methods

The data were analyzed via SPSS 25.0 software. The measurement data were presented as the means (standard deviations), and the count data were presented as frequencies (percentages). For normally distributed data, independent samples *t*-tests were conducted for two-group comparisons, whereas one-way ANOVA was used for three or more groups. When ANOVA results were significant, pairwise comparisons were performed via the least significant difference method to assess differences in SMB among the groups. If any main variables showed significant differences between groups, the grouping was included as a control variable in the mediation regression model. Pearson correlation coefficients were calculated for correlation analysis between variables. Additionally, chained mediation analysis was performed using PROCESS v3.3 (Model 6), which was developed by Hayes (2009), with 5000 bootstrap random samples. A significance level of *P* < 0.05 was adopted.

## 3 Results

### 3.1 Participant characteristics and SMB differences

A total of 198 questionnaires were collected, 183 of which were valid, and 15 invalid questionnaires were excluded due to the regularity of responses and age over 24 years. The effective recovery rate was 92%. The participants distribution in this study was as follows: East China (30.05%), North China (2.73%), Central China (7.10%), South China (6.55%), Southwest China (49.18%), and Northeast China (4.37%). The participants had a mean age of 20.33 ± 3.03 years, with an average SMB score of 137.73 ± 20.62 (out of 180).

Table 1 summarized the differences in SMB scores across participant characteristics. *T-*tests and ANOVA analyses revealed significant differences in SMB scores based on age and disease duration (*P* < 0.05). Younger participants had higher SMB scores (141.31 ± 20.87) than older participants did (134.76 ± 20.04). Moreover, those with a disease duration >3 years had significantly higher SMB scores (143.40 ± 20.00) than those with a duration of ≤ 1 year (132.74 ± 16.33) and 1–3 years (136.33 ± 21.65), with significant differences (*P* < 0.05). No significant differences were found between the ≤ 1-year and 1–3-year groups (*P* > 0.05).

**Table 1:** Differences in SMB among participants with different characteristics (*N* = 183).

	**Frequency (percentage)**	**SMB**	
		***t/F***	***P***
**Age (years)**		**2.161** ^a^	**0.032** ^*^
13–20	83 (45.36)		
21–24	100 (54.64)		
**Gender**		**0.970** ^a^	**0.333**
Male	131 (71.58)		
Female	52 (28.42)		
**Residence**		**0.411** ^a^	**0.681**
Urban	119 (65.03)		
Rural	64 (34.97)		
**Annual household income (RMB)**		**0.987** ^b^	**0.400**
≤ 20,000	27 (14.75)		
20,001–50,000	65 (35.52)		
50,001–150,000	71 (38.80)		
>150,000	20 (10.93)		
**Education background**		**0.438** ^b^	**0.726**
Middle school	19 (10.38)		
High school	33 (18.03)		
College	87 (47.54)		
Post-college	44 (24.04)		
**Disease type**		**0.363** ^a^	**0.717**
CD	163 (89.07)		
UC	20 (10.93)		
**Disease duration**		**3.179** ^b^	**0.044** ^*^
< 1	31 (16.94)		
1–3	100 (54.64)		
>3	52 (28.42)		
**Have undergone IBD-related surgery**		−**0.308**^a^	**0.759**
Yes	59 (32.24)		
No	124 (67.76)		
**Medications categories**		**0.294** ^a^	**0.769**
1	115 (62.84)		
≥2	68 (37.16)		

^a^*t* value; ^b^*F* value; ^*^P < 0.05.

### 3.2 Correlations between the main variables

Pearson correlation analysis was performed between perceived social support, basic psychological needs, anxiety and depression, and SMB. The results, as shown in Table 2, revealed a positive correlation between perceived social support, basic psychological needs, and SMB (*r* > 0, *P* < 0.001) and a negative correlation between perceived social support, basic psychological needs, SMB and anxiety/depression (*r* < 0, *P* < 0.001).

**Table 2:** Correlations between SMB, perceived social support, basic psychological needs, anxiety and depression (*N* = 183).

	***M*(*SD*)**	**SMB**	**Perceived social support**	**Basic psychological needs**	**Anxiety**	**Depression**
SMB	137.73(20.62)	1				
Perceived social support	63.50 (13.25)	0.637^***^	1			
Basic psychological needs	48.31 (9.75)	0.658^***^	0.769^***^	1		
Anxiety	5.39 (5.15)	−0.527^***^	−0.572^***^	−0.587^***^	1	
Depression	5.74 (5.58)	−0.556^***^	−0.585^***^	−0.585^***^	0.860^***^	1

^***^P < 0.001.

### 3.3 Chained mediation model analysis

A chain mediation effect model was constructed with perceived social support as the independent variable, basic psychological needs and anxiety/depression as the mediator variables, SMB as the dependent variable, and age and disease duration as the control variables. Regression analysis showed that perceived social support significantly positively predicted basic psychological needs (β = 0.767, *P* < 0.001) and significantly negatively predicted anxiety and depression (β = −0.278, *P* < 0.01; β = −0.314, *P* < 0.01), and basic psychological needs significantly negatively predicted anxiety and depression (β = −0.356, *P* < 0.001; β = −0.328, *P* < 0.001). When perceived social support, basic psychological needs, anxiety, and SMB were included in the regression model, perceived social support still had a significant direct effect on SMB (β = 0.261, *P* < 0.01); basic psychological needs and anxiety had a significant predictive relationship with SMB (β = 0.356, *P* < 0.001; β = −0.148, *P* < 0.05). When perceived social support, basic psychological needs, depression, and SMB were entered into the regression model, perceived social support still had a significant direct effect on SMB (β = 0.241, *P* < 0.01); basic psychological needs and depression had a significant predictive relationship with SMB (β = 0.345, *P* < 0.001; β = −0.194, *P* < 0.01). See Table 3 for details.

**Table 3:** Results of regression analysis between SMB, perceived social support, basic psychological needs, and anxiety/depression.

**Result variables**	**Predictive variables**	***R^2^***	***F***	**β**	***t***	***P***
Basic psychological needs	Perceived social support	0.592	86.418	0.767	15.784	< 0.001
Anxiety	Perceived social support	0.395	29.021	−0.278	−3.028	0.003
	Basic psychological needs			−0.356	−3.896	< 0.001
SMB	Perceived social support	0.503	35.772	0.261	3.054	0.003
	Basic psychological needs			0.356	4.120	< 0.001
	Anxiety			−0.148	−2.177	0.031
Depression	Perceived social support	0.398	29.376	−0.314	−3.437	0.001
	Basic psychological needs			−0.328	−3.600	< 0.001
SMB	Perceived social support	0.512	37.127	0.241	2.830	0.005
	Basic psychological needs			0.345	4.057	< 0.001
	Depression			−0.194	−2.864	0.005

Table 4 demonstrated that when the chained mediator variables were basic psychological needs and anxiety, basic psychological needs partially mediated the relationship between perceived social support and SMB (95% CI: 0.204–0.630), and anxiety mediated the relationship between perceived social support and SMB (95% CI: 0.000–0.162), and the chain of basic psychological needs and anxiety between perceived social support and SMB mediating role was also significant (95% CI: 0.006–0.152). The total effect value was 0.958, the total direct effect value was 0.406, and the total indirect effect value was 0.552, representing 57.60%.

**Table 4:** Mediation effect tests for basic psychological needs and anxiety in perceived social support and SMB.

	**Effect value**	***SE***	**Bootstrap 95% CI**	**Relative mediation effect (%)**
Total indirect effect	0.552	0.110	[0.338, 0.773]	57.60%
Perceived social support → basic psychological needs → SMB	0.425	0.105	[0.204, 0.630]	44.33%
Perceived social support → anxiety → SMB	0.064	0.043	[0.000, 0.162]	6.69%
Perceived social support → basic psychological needs → anxiety → SMB	0.063	0.038	[0.006, 0.152]	6.56%

Table 5 demonstrated that when the chain mediators were basic psychological needs and depression, basic psychological needs partially mediated the relationship between perceived social support and SMB (95% CI: 0.190–0.597), and depression partially mediated the relationship between perceived social support and SMB (95% CI: 0.012–0.206), and the chain mediation of basic psychological needs and depression between perceived social support and SMB was also significant (95% CI: 0.015–0.181). The total effect was 0.958, with a total direct effect value of 0.375 and a total indirect effect value of 0.583, accounting for 60.80%.

**Table 5:** Mediation effect tests for basic psychological needs and depression in perceived social support and SMB.

	**Effect value**	***SE***	**Bootstrap 95% CI**	**Relative mediation effect (%)**
Total indirect effect	0.583	0.110	[0.363, 0.801]	60.80%
Perceived social support → basic psychological needs → SMB	0.412	0.102	[0.190, 0.597]	43.00%
Perceived social support → depression → SMB	0.095	0.050	[0.012, 0.206]	9.89%
Perceived social support → basic psychological needs → depression → SMB	0.076	0.044	[0.015, 0.181]	7.91%

**Figure 1:**
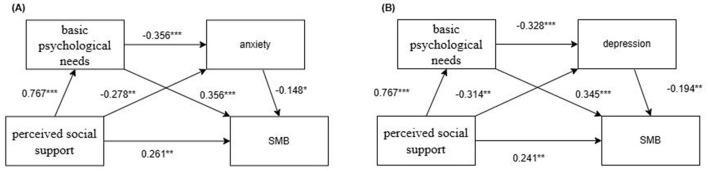
Chained mediation model of perceived social support affecting SMB. **(A)** Basic psychological needs and anxiety as mediator variables. **(B)** Basic psychological needs and depression as mediator variables. ***P < 0.001, **P < 0.01, *P < 0.05.

These results suggested that perceived social support could influence SMB through the chain mediation of basic psychological needs and anxiety/depression (see Figure 1).

## 4 Discussion

In this study, we found that the SMB scores of the older age group among participants were lower than those of the younger age group, which stood in contrast to existing surveys (Foster et al., 2023) that reported a positive correlation between age and readiness for the transition to adulthood. This discrepancy may stem from parental overprotection, especially in Asia, where younger adolescents tend to be strongly constrained by their parents and tend to rely on their families for self-management. As these adolescents gradually seek independence, their ability may not be sufficient to maintain good SMB status. Therefore, this finding further emphasized the importance of developing independent self-management skills in adolescents and young adults with IBD at an early age.

We examined the relationships among perceived social support, basic psychological needs, and SMB in a population of adolescents and young adults with IBD. Our results indicated that higher levels of perceived social support were associated with better SMB in these participants, a finding that aligned with a previous study (Kamp et al., 2019b). Additionally, basic psychological needs was found to partially mediate the relationship between perceived social support and SMB, supporting our first research hypothesis. Given these findings, it is crucial for intervention studies targeting SMB in adolescents and young adults with IBD to focus on enhancing both perceived social support and basic psychological needs. Researchers (Roberts et al., 2021; Tan and Ong, 2020) indicated that adolescents and young adults with IBD often experience peer victimization at school and experience overprotective parenting at home. These experiences could contribute to insufficient perceived social support and unmet basic psychological needs for them. To address this issue, clinical practice should promote collaboration between schools and families to enhance social support and fulfill basic psychological needs, thereby facilitating positive SMB in adolescents and young adults with IBD. As adolescents mature, the influence of peers tends to surpass that of parents (Adriano et al., 2022). Therefore, interventions that incorporate peer support may prove to be more effective, as supported by previous research (Adriano et al., 2022). The incorporation of patient organizations could be a valuable resource for peer support, and clinical staff should assist adolescents and young adults with IBD in connecting with local patient organizations. Furthermore, increasing social support must be paired with efforts to enhance adolescents' perceptions of the available support. This find aligned with the solution-focused approach (McAllister, 2003), which emphasizes the importance of actively exploring and utilizing one's resources. Future intervention studies could leverage the solution-focused approach to enhance the perceived social support and satisfaction of basic psychological needs among adolescents and young adults with IBD, ultimately improving their SMB.

This study found that anxiety and depression partially mediated the relationship between perceived social support and SMB, supporting the second research hypothesis. Anxiety and depression emerged as significant negative predictors of SMB, aligning with a previous finding (Cao et al., 2023). Interestingly, a study (Distaso et al., 2022) indicated that avoidant coping behaviors characteristic of anxiety syndrome could promote SMB in adults with diabetes. Future research should conduct a characteristic analysis of SMB among adolescents and young adults with IBD, as different characteristics of SMB may be influenced by variables through distinct pathways. However, considering the developmental stage of adolescents and young adults, who often lack the emotional regulation skills necessary to transform negative emotions into motivational incentives for effective SMB, clinicians should address their common emotional issues, specifically anxiety and depression. We found that enhancing perceived social support among adolescents and young adults with IBD could contribute to alleviating anxiety and depression, which was consistent with the buffering theory of social support (Miloseva et al., 2017). Furthermore, mindfulness interventions have been shown promise in alleviating anxiety and depression among adolescents and young adults with IBD (Hughes et al., 2023). Mindfulness emphasizes awareness and attention to the present moment (Hughes et al., 2023), which could also potentially improve individuals' perceptions of social support.

Our findings indicated that perceived social support could affect SMB through a chain mediation involving basic psychological needs and anxiety/depression, thereby substantiating the third hypothesis. This finding supported self-determination theory (Ryan and Deci, 2000), which posited that social support could foster the satisfaction of basic psychological needs, enhance individuals' wellbeing, and alleviate symptoms of anxiety and depression. Consequently, adolescents and young adults were better positioned to adopt effective SMB. Researchers (Gohil et al., 2022; Tran and Mulligan, 2019) suggested that the most effective strategy for improving SMB in adolescents and young adults with IBD was multicomponent, which was consistent with our findings that multiple factors and pathways could influence SMB. In conclusion, clinical staff should integrate health education and psychological techniques to implement comprehensive interventions that address perceived social support, basic psychological needs, anxiety, and depression to optimize SMB in adolescents and young adults with IBD.

Building upon existing literature, this study further elucidated the pathway through which perceived social support influenced SMB in adolescents and young adults with IBD, which was mediated by basic psychological needs and anxiety/depression. The insights garnered from this research could hold implications for the practical design of interventions to improve SMB of adolescents and young adults with IBD.

## 5 Limitations

This study had several limitations. First, due to the cross-sectional design employed in this study, causal relationships between perceived social support, basic psychological needs, anxiety, depression, and SMB could not be determined; thus, longitudinal studies are needed to explore these relationships more comprehensively. Second, this study was conducted during the summer vacation period, and the possible effects of vacations on the psychology of adolescents and young adults with IBD could be explored again in future studies during non-vacation periods to explore the variable relationships. Third, the gender balance in our sample requires improvement, as the prevalence of IBD is higher in males than in females in China (Xu et al., 2023). Future research should consider the gender balance to ensure more representative findings. Fourth, the study's data relied on self-reported questionnaires. Future research could incorporate objective indicators to enhance the objectivity of the findings. Additionally, confirming the IBD diagnosis through self-report in participants recruited via the snowball method represents a limitation of the study. Lastly, participants with comorbid psychiatric diagnoses were excluded. Considering the increasing prevalence of mental disorders among patients with IBD, future research could include this population to gain a more holistic understanding of the dynamics at play.

## 6 Conclusion

This study identified perceived social support as a predictor of SMB in adolescents and young adults with IBD. Furthermore, basic psychological needs, as well as anxiety and depression, were found to mediate the relationship between perceived social support and SMB. These findings enhanced our understanding of the mechanisms driving the development of SMB in this population, which was crucial for facilitating a smooth transition to adulthood, improving quality of life, and even influencing disease prognosis. Consequently, strategies aimed at increasing social support, improving perceptions of social support, fulfilling basic psychological needs, and alleviating anxiety and depression should be prioritized to promote SMB among adolescents and young adults with IBD.

## Data Availability

The original contributions presented in the study are included in the article/supplementary material, further inquiries can be directed to the corresponding author.
